# Burning Questions

**Published:** 1894-12-29

**Authors:** 


### The Hospital

An Institutional Journal of

The Medical Sciences and Hospital Administration, with

Vol. XVII.—No. 431.] AN EXTRA NURSING SUPPLEMENT. [Dec. 29, 1894.

### Burning Questions.

It will hardly be denied by those who know, that medicine has become a "learned profession" on a much wider scale than either of the other professions of learning, the Church or the Law. The proportion among us of persons who are widely and deeply read, whose learning is what Mr. Gladstone has in another connection called "massive," is very large, whilst the so-called "rank and file" of the profession are only "rank and file" in the sense that their work is all-round work and their emoluments are poor. For general culture and for special technical capacity in their art, the general practitioners of medicine are beyond comparison superior to the general practitioners of either of the other learned professions. Upon these undoubted facts we base the proposition that since medicine consists so largely of really learned persons it ought as a calling to be made such in all its details as to be worthy of the loyal allegiance of those whose lives have been devoted to it.

In actual practice medicine, except perhaps for one in a score, is neither an agreeable, nor a profitable, nor a healthy occupation. On the contrary, it is harassing beyond measure, and brings many men to early graves and their families to poverty. It has often been said that these things ought not so to be. Let it now be said that these things shall not so be. The close of the year is a favourable time for taking the measure of the present situation, and for considering whether progression or retrogression has been the prevailing note of the past year in medical politics and ethics. One thing, at any rate, we may congratulate ourselves upon. It has been a year of life, of universal and eager activity. The general practitioner is very much awake, whoever else may be sleeping. For proof of this we have but to recollect such facts as the recent decision of the General Medical Council, impelled by a section of general practice, on the question of the private certification of midwives; the strenuous action of almost the whole of the practitioners of North London in connection with the recent opening of pay wards at the Great Northern Central Hospital; the dead set made against unqualified practice by medical associations; the keen prosecution of coverers; the almost universal movement in favour of an increase of medical and surgical fees; and the determination to make life intolerable for all those unwholesome and injurious practitioners who, in their eagerness to be surrounded by crowds of patients, pursue methods of competition worthier of the travelling showman than of educated members of a scientific profession. All these are indications of professional virility and activity which the dullest cannot deny.

Some of the questions we have indicated are of the first importance, more especially to the younger members of the profession who have elected to follow general practice. Pre-eminent among these is the question raised so resolutely by the profession in North London of the entry of the general practitioner into the wards of the hospital in order to the better success of his practice. It is now universally recognised that a well-equipped hospital has certain advantages over the homes of the less wealthy members of the middle classes for the treatment of cases of serious and prolonged illness where skilled and unremitting attention to medical details is almost the most important part of the technique of treatment, and where the lack of such unremitting attention may be the one factor which turns the scale against the patient.

Shall family physicians, when they perceive that skilled nursing with other hospital advantages are indispensable for their patient's welfare, be entitled in suitable cases to order removal to hospital, and there to continue in charge of their own patients, to the vital advantage of those patients, or shall they not? The question, it will be seen, is one of very great moment, not to family doctors only, but to their patients; and not alone to these two classes, but to every class and condition of the whole population. We do not presume to lay down any definite judgment upon the matter offhand; but we do say, and urge insistently upon the profession, that it is a question of the very first importance to us all, and a question the discussion of which will hardly brook further delay.

The subject of the education and registration of midwives is one of those standing dishes in medical politics which are the opprobrium of the profession and which make outsiders mount the seat of the scornful. It is plain that the country needs midwives; it is equally plain that the medical profession is honourably bound to protect it against incompetent midwives, and also to supply it with those who are competent. How is this to be done? That is the simple question at issue. It is true that all sorts of side issues are raised, by all sorts of persons, for all sorts of purposes. But the plain issue is this, and nothing else: Does the country need midwives? And if it does, who but the medical profession is competent to furnish them? If that be so, and it is, then we ought to be ashamed that the sole result of years of agitation is a miserable *impasse* and deadlock. The medical world owes it to its own reputation and general culture to cut its way through all the unworthy entanglements which mistaken interests have surrounded it with, and to act in the interests of the public and of itself with statesmanlike judgment and decision.

The questions of unwholesome competition, of covering, of unqualified practice, of fees so low as to degrade even the lowest classes of unskilled labourers, are clearly practical factors in the daily life of medicine which must determine, and in no long time, either its rise or its fall. It is impossible that they can exist without exerting a potent effect upon all those who are directly or indirectly under their influence; that is, of course, upon the whole profession. They demand for their solution two things: The highest practical statesmanship, and the utmost possible dis interestedness on the part of those who would solve them. There are great opportunities here for competent leaders. Well may medicine, like the Pagans in the shipwreck, call upon its gods for help and deliverance.

